# Early introduction of simulation in the medical curriculum: the MedInTo perspective

**DOI:** 10.3389/fmed.2023.1280592

**Published:** 2024-01-04

**Authors:** David Lembo, Federico Abate Daga, Corrado Calì, Diego Garbossa, Matteo Manfredi, Lorenzo Odetto, Luca Ostacoli, Piero Paccotti, Stefania Raimondo, Giuseppe Reimondo, Savino Sciascia

**Affiliations:** MD Program in Medicine and Surgery of University of Turin-MedInTo, Department of Clinical and Biological Sciences, University of Turin, Turin, Italy

**Keywords:** simulation, medical education, medical curriculum development, medical student, virtual reality

## Abstract

Despite the increasing body of evidence supporting the use of simulation in medicine, a question remains: when should we introduce it into the medical school's curriculum? We present the experience and future perspectives of the MD program in Medicine and Surgery of University of Turin-MedInTo. Since its launch, MedInTo has been dedicated to integrating innovative teaching approaches at the early stages into the medical curriculum. Herewith, we describe a case-based approach for our activities, which includes the utilization of simulation for emergency medical care training for students and the integration of virtual and augmented reality technology. Dedicated surgical training activities using virtual-augmented reality and life-like simulator for students are also described.

## Introduction

The early introduction of simulation in the medical curriculum has become increasingly prevalent in recent years (1). Simulation refers to the use of realistic scenarios and equipment to replicate clinical situations for educational and training purposes (2). Integrating simulation into medical education offers several benefits, including enhanced learning experiences (3), improved clinical skills (4–6), and increased patient safety (2, 6).

Evidence supporting the use of simulation learning to deliver medical training is available across all grades and specialties, ranging from surgery (7, 8), including robot surgery (9), infectious diseases, pediatrics, orthopedics (10), and internal medicine (11–15).

However, despite the increasing body of evidence supporting the use of simulation in medicine, a question remains: when should we introduce it into the medical curriculum? Is an early approach feasible?

Aiming to contribute to this topic, we report the experience and future perspectives of the MD program in Medicine and Surgery of University of Turin-MedInTo. Since its launch, MedInTo has been devoted to integrating innovative teaching approaches into the medical curriculum. Entirely taught in English, MedInTo takes place in the teaching facilities at San Luigi Gonzaga University Hospital (Orbassano, Torino, Italy), whose campus-type environment aims to optimize the integration of students with the clinical areas. Since early in the degree program, the curriculum combines scientific and clinical knowledge with interactive teaching methods. The educational goals of our degree program are particularly suited for students interested in scientific research and cooperation. The proximity of teaching, research, and hospital facilities promotes communication between students and teaching staff, as do the integrated courses and the practice-based learning opportunities.

When referring to the early introduction of simulation, here are some key points summarizing the conceptual framework on which the MedInTO has been designed.

- **Experiential learning**: Simulation provides students with hands-on, experiential learning opportunities that bridge the gap between theory and practice (16). By engaging in realistic scenarios, students can apply their knowledge and develop critical thinking, clinical reasoning, and decision-making skills in a safe and controlled environment (17).
- **Skill acquisition**: Simulation-based training allows students to practice and refine their technical skills before working with real patients. They can learn and practice procedures such as suturing (18), phlebotomy (19, 20), or catheter insertion (21) on high-fidelity manikins or virtual reality simulators. This early exposure helps students gain proficiency and confidence in performing these skills (22, 23).
- **Teamwork and communication**: Healthcare is a collaborative field that requires effective teamwork and communication among healthcare professionals. Simulation scenarios involving interprofessional teams provide opportunities for students from various disciplines (e.g., medicine, nursing, pharmacy) (24) to learn and practice teamwork (25), communication, and coordination skills (26). They can learn how to communicate effectively, delegate tasks (27), and work together to provide optimal patient care (28). This approach has the potential to support the identification of further educational needs based on the experience of the clerkship (29).
- **Mistake management and patient safety**: Simulation offers a safe environment to make and learn from mistakes without compromising patient safety. Students can encounter challenging clinical situations, experience the consequences of their actions, and receive immediate feedback (30) from instructors (31). This iterative learning process helps develop clinical judgment, error recognition, and error management skills, thereby reducing the risk of errors and enhancing patient safety (6, 32).
- **Ethical and complex scenarios**: Simulation allows students to engage with ethical and complex scenarios that may be challenging to encounter in real-life clinical settings (33). This includes navigating dilemmas (34), making ethical decisions, and engaging in difficult conversations with patients and their families. Simulation provides a platform to reflect on the ethical dimensions of healthcare, fostering the development of empathy and professionalism (33).
- **Bridging the gap between classroom and clinical practices**: Early exposure to simulation can help ease the transition from the classroom to clinical practice (35, 36). By familiarizing students with realistic patient cases, medical technology, and clinical environments, simulation can reduce anxiety (37) and enhance students' preparedness and confidence when interacting with real patients (35, 38).
- **Research and innovation**: Simulation-based education provides opportunities for research and innovation in medical education. It allows educators to develop and evaluate new teaching methods, curricula, and assessment tools (39, 40). Additionally, advancements in technology, such as virtual reality (41) and augmented reality (42), continue to expand the possibilities of simulation-based training in healthcare education (43).
- **Economization of resources**: Early exposure to simulated scenarios and/or dummy instrumentation allows for maximizing the cost-benefit balance between early skill acquisition while reducing the economic and human costs of medical teaching and learning (44–47).

The above-mentioned points constitute the conceptual framework supporting the design of education activities aimed at optimizing the integration of simulation into the medical MD curriculum, especially in an early phase. A summary of ongoing or upcoming activities is listed below.

### Simulation for emergency medical care for students

This learning experience aims to help the students understand the basis of emergency medical services. Emergency medical care is characterized by interventions that are done “outside the hospital,” where the environment or evolution of risk deeply influences the intervention patterns. For these reasons, this training is crucial for students who are approaching the completion of their medical studies career. Thus, we employed the “learning by doing and learning by feeling” (48) methodology to guide them in thinking and acting as real EMC operators.

The experience is made both in the simulation center and in the nearby environment to empower the simulation alternating structured and eco-dynamic environments.

**Figure 1:**
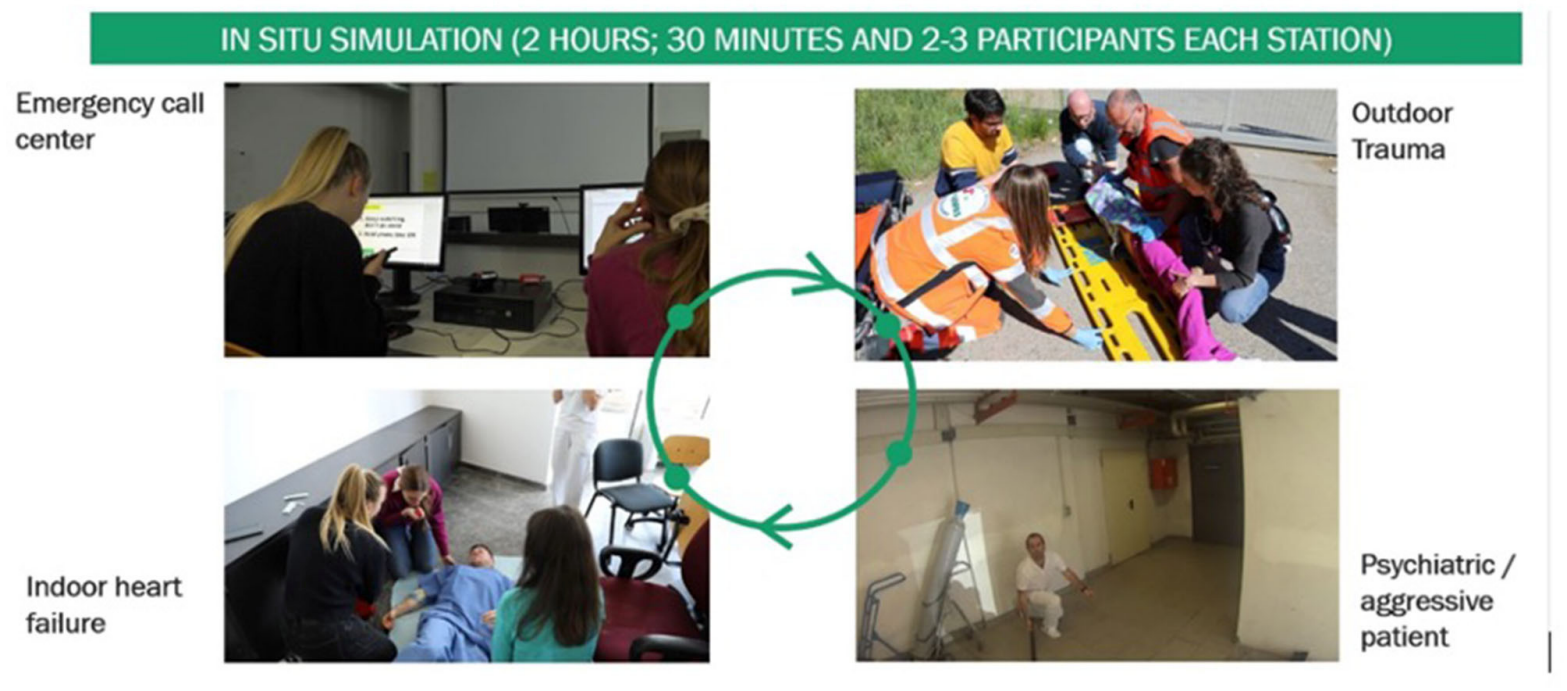
Schematic summary of the simulation for emergency medical care for students' activity.

After 1 h of technical skill learning (patient approach, skill acquisition, etc.), students were inserted in the “simulation circuit” (Figure 1). In both the center and nearby environments, four contemporaneously simulative stations were organized (Supplementary Video). Students approached four different cases: Managing a simplified emergency call center, an indoor heart failure, handling a psychiatric-aggressive patient, and addressing an outdoor trauma. Each station was 30 min long and students rotated through them until the routine was completed. A final debriefing was carried out by the trainer at the end of each station.

## Anatomy first: the anatomage table

Basic disciplines, such as human anatomy, form the core subjects in medical and health science education. They are crucial for understanding the functioning of the human body and providing a basis for clinical training and practice. The most ancient and prestigious medical schools have a tradition of hands-on human body dissection, leading to the establishment of institutes dedicating to normal human anatomy, where human cadavers were stored and dissected by teachers and medical students. As powerful as this approach is, the availability of human cadavers, the legislation, and the costs related to their maintenance make it less accessible to a large number of students (49). For this reason, thanks to technological advancements and the increased use of 3D modeling, an increasing number of interactive 3D atlases are now accessible, with the vast majority being available online. The first coordinated effort to produce an interactive 3D atlas based on real data was the 1986 “Visible Human Project,” leading to the dissection of a full human cadaver into 4 mm thin slices, each meticulously photographed and segmented. Additionally, MRI and CT scans were incorporated to better highlight the visualization of different structures. The results were first published in 1994. Recently, a US-based company, Anatomage, developed a state-of-the-art device, featuring four fully segmented interactive cadavers, cut at a higher resolution (up to 0.8 mm). These digital cadavers are loaded onto a dissection-sized touchscreen table that can be used for performing a virtual dissection. This device is now present in a significant number of anatomy institutions all over the world, and although it cannot fully replace hands-on dissection, it is a very powerful teaching device for medical students at the early stages of their education (50).

To date, the Anatomage table has been introduced in anatomy teaching for undergraduate medical and nursing students in the MedInTO course. It is used both for teacher-directed learning and self-dependent study learning. In our experience, we highlighted that Anatomage enhanced active learning by allowing the students to clarify doubts and share their anatomical knowledge, thereby improving communication skills.

Moreover, the Anatomage table can be used for many different applications. It not only displays preloaded 3D datasets but also functions as a powerful DICOM viewer with automated rendering capabilities and high GPU specs, making it suitable for personalized medical applications, including diagnosis and surgical planning. Additionally, it proves invaluable in teaching on real datasets using non-invasive imaging techniques. Recently, one interesting application has been proposed to use it as a virtual autopsy device, with a special focus on dental analysis for disaster victim identification (51). The presence of this device in the simulation facility serves as a perfect companion to help students revise the real anatomical reference related to procedures they might be testing on real field.

## Virtual and augmented reality

Virtual and Augmented reality (VR and AR) are relatively old technologies, with their early appearance in the late 50s and late 60s of the last century, respectively. Although VR originally started with a leisure scope (“Sensorama” was the grandfather of immersive 3D cinema), this technology has a natural declination for testing and simulation in nature (8). The high cost of these devices made this technology accessible to institutions and high-end companies, primarily utilizing them for medical, flight, and automotive simulations. The remarkable and rapid advancements in computer graphics technology, possibly linked to the increased efficiency of GPU data processing in machine learning (ML) and artificial intelligence (AI), has massively contributed to popularize this technology. This technology is affordable to the mass consumer market, consequently making it more accessible to research institutions and small companies with a focus on R&D (52, 53). AR has also benefited a lot from improvements in Computer Vision (CV) field, with the commercialization of head-mounted AR devices allowing 3D stereoscopic view of digital objects and the possibility of interacting with those, even with bare hands. Interactive AR is also called mixed reality (MR or XR). The potential of these technologies can be limited only by imagination, and medical simulation is already harnessing significant advantages from them (53–55). VR can be easily used to recreate various medical scenarios, including emergency rooms, operating rooms, ambulances, and even consulting rooms. The possibility of interacting with the scenes gives a perfect opportunity to develop flexible platforms that allow multiple scenarios with only one computer and a headset. It is also possible to train specific skills, for instance, in surgery, using haptic devices that simulate scalpels or other surgical instruments, and it can simulate the interaction with skin and other deeper tissues, using a force feedback logic.

Another example of virtual reality is the 3D virtual reconstructions of organs (56). In the surgical field, they have proved to be valid tools in different settings, including aiding in surgical indications—helping the surgeon to choose, for example, between a radical or a partial surgery—and assisting in the decision-making process preceding surgery (i.e., surgical planning). Additionally, they serve as valuable tools in surgical training; and intraoperative navigation (57).

Augmented and mixed reality are increasingly used in medical simulation, to digitally enrich the visual perception of users (students, residents, and medical doctors), representing a powerful aid to medical simulation. For instance, with the aid of computer vision, it is possible to map the environment and the hands of students to trace their ability to perform certain skills. Additionally, this technology enables the superimposition of deeper anatomical structures (e.g., vascularization, major nerves) to train performing safely minimally invasive procedures.

In robot-assisted surgery (58), for example, 3D virtual reconstructions can be presented, handled, and modified according to the surgeon's needs and sent into the robotic console via software, reproducing the real surgical procedure and simulating the different surgical steps.

Finally, the use of mixed approaches with XR-enhanced phantoms or mannequins represents a powerful tool compromising the flexibility of a VR scene with the training on a real object, designed to give the user an experience as similar as possible to the real field. A startup company “Intravides,” from the University of Turin, which has collaborated with neurosurgery planning and remote assistance, has now begun to work in the safer environment of a simulation room to plan minimally invasive surgery.

A more expensive, yet powerful, solution is the immersive technology, which allows you to project any sort of scenario in a real room, with the possibility of interacting with the digital content. The possibility of interacting and blending a real environment with a digitally enhanced one brings it closer to XR, although this kind of technology is often referred to as VR.

### Virtual reality technology for doctor–patient communication

To effectively educate and train medical students in the essential skill of doctor–patient communication, a comprehensive program was implemented, utilizing virtual reality technology and a virtual campus. The program was developed through a collaboration between the School of Medicine and Surgery, Department of Clinical and Biological Sciences, University of Turin, and iGoOver Srl (www.igoover.it), a company specializing in experiential training using innovative methodologies and technologies. Thanks to a 2-year agreement, research and didactic training were carried out. Additionally, over 50% of the students in the course were from European and non-European countries.

Virtual reality immersion training is an effective teaching method for fostering empathy among students in medical and health professions (59, 60). This innovative approach aimed to provide students with practical experiences and valuable insights into communication strategies in emotionally charged medical scenarios. The program comprised two informative sessions that focused on different aspects of effective communication.

To ensure the authenticity and relevance of the training, four carefully crafted clinical cases were developed in collaboration with experienced oncologists. Each case had distinct communication goals, addressing various challenging scenarios. In the first case, the objective was to convey a diagnosis and discuss the need for surgery, emphasizing the importance of postoperative follow-up for accurate assessment. The second case involved communicating the results of a histological exam, explaining the necessity of chemotherapy to prevent relapse, and discussing potential side effects. The third case centered around a patient's deteriorating condition during chemotherapy, requiring the delicate task of conveying the decision to transition from active therapy to supportive care. In the fourth case, students were tasked with persuading a patient who had undergone surgery to accept a more aggressive treatment plan, emphasizing the ongoing presence of the disease.

To enhance the realism of the training, professional actors were enlisted to portray the patients, enabling students to engage in authentic interactions during consultations. This approach allowed students to develop their communication skills through direct patient interaction and by observing doctor–patient communication in the plenary room. Recent studies found that prior exposure to simulation training, such as communication skills, medical humanistic care, and checklist completion in subsequent asthma exacerbation simulation-based training, can improve the performance of medical students (61, 62).

Following each session, dedicated time was allocated for discussion and debriefing, facilitating an open dialogue where students could reflect on their strengths and areas requiring improvement. This critical reflection period provided students with the opportunity to analyze and refine their communication abilities, resulting in enhanced proficiency and confidence.

Furthermore, in addition to the practical training, the program incorporated mindfulness-based concepts to equip students with valuable tools for managing emotions during challenging medical communications. A recent systematic review on the impact of mindfulness-based interventions on doctors' wellbeing and performance (63) reported how doctors exposed to mindfulness-based interventions exhibited lower levels of negative wellbeing (burnout, stress, and anxiety) and higher levels of positive wellbeing (empowerment, dedication, and satisfaction). The authors highlighted that patients may, indirectly, benefit from mindfulness-based interventions because doctors who had undertaken such interventions were reported to provide more empathic and patient-centered care.

By integrating these innovative approaches, the Medicine and Surgery degree program demonstrated its commitment to preparing future medical professionals with the necessary expertise in doctor–patient communication.

The success of this pioneering initiative showcased the effectiveness of utilizing virtual reality technology, realistic clinical cases, and dedicated reflection periods, solidifying the program's impact on nurturing well-rounded and proficient medical practitioners.

## Virtual-augmented reality and life-like neurosurgical simulator for training: evaluating a hands-on experience for residents and students

In the recent years, growing interest in simulation-based surgical education has led to various practical alternatives for medical training. More recently, courses based on virtual reality (VR) and three-dimensional (3D)-printed models have become available. We suggest a hybrid (virtual and physical) neurosurgical simulator that has been validated, equipped with augmented reality (AR) capabilities that can be used repeatedly to increase familiarity and improve the technical skills in human brain anatomy and neurosurgical approaches. This method could be used not only in neurosurgical training but also in certain aspects of some general surgical training for medical students.

New, high-fidelity, cadaver-free simulators “UpSurgeon” together with other devices, such as Google glass for augmented reality and a camera for remote acquisition and PC vision, provide an opportunity to increase access to skills laboratory training and operative room tutoring. Surgical skills laboratories augment educational training by deepening one's understanding of anatomy and allowing the safe practice of technical skills. The neurosurgical field has historically evaluated skills by subjective assessment or outcome measures, as opposed to process measures with objective, quantitative indicators of technical skill and progression. We have designed a pilot training schedule with spaced repetition learning concepts to evaluate its feasibility and impact on proficiency. Then, we could observe the different steps of surgical procedures in videos, interact with surgeons, and share the images with other experts for the evaluation of the surgery. Additionally, these images could be shared with students or residents for teaching purposes. The 6-week module used (2) macrosuture-microsuture, (3a) bone simulator for drilling, (3b) endoscopic maneuvers exercises with fruit (basic activities for resident and MD students), (4) a simulator of a pterional, suboccipital, interhemispheric, endoscopic endonasal approaches representing the skull, dura mater, cranial nerves, and arteries, (5) simulator of surgical clipping, tumor resection (UpSurgeOn S.r.l.). Neurosurgery residents and students completed a video-recorded baseline examination, performing different craniotomies, dural opening, suturing, and anatomical identification. These were recorded using a smart phone and or under a microscope. Participation in the full 6-week module was entirely voluntary. In the 6th week, all residents and students repeated the initial examination, which was recorded on video. Videos were evaluated by three neurosurgical attendees who were not affiliated with the institution and who were blinded to participant grouping and year. Scores were assigned via global rating scales (GRSs) and task-based specific checklists (TSCs) previously built for craniotomy (cGRS, cTSC) and microsurgical exploration (mGRS, mTSC). Participants who underwent a 6-week simulation course showed significant objective improvement in technical indicators, particularly individuals who were early in their training. Small, non-randomized grouping limits generalizability regarding the degree of impact; however, introducing objective performance metrics during spaced repetition simulation would undoubtedly improve training. A larger multi-institutional randomized controlled study will be shared to elucidate the value of this educational method.

The hybrid AR and 3D-printed neurosurgical simulator could be a valid tool for neurosurgical training, capable of enhancing personal technical skills and competence. In addition, it could be easy to imagine how patient safety would increase and healthcare costs would be reduced, even if more studies are needed to investigate these aspects. The integration of simulators for training in neurosurgery as preparatory steps for the operating room should be recommended and further investigated given their huge potential.

## Laparoscopic pelvic box trainers

**Figure 2:**
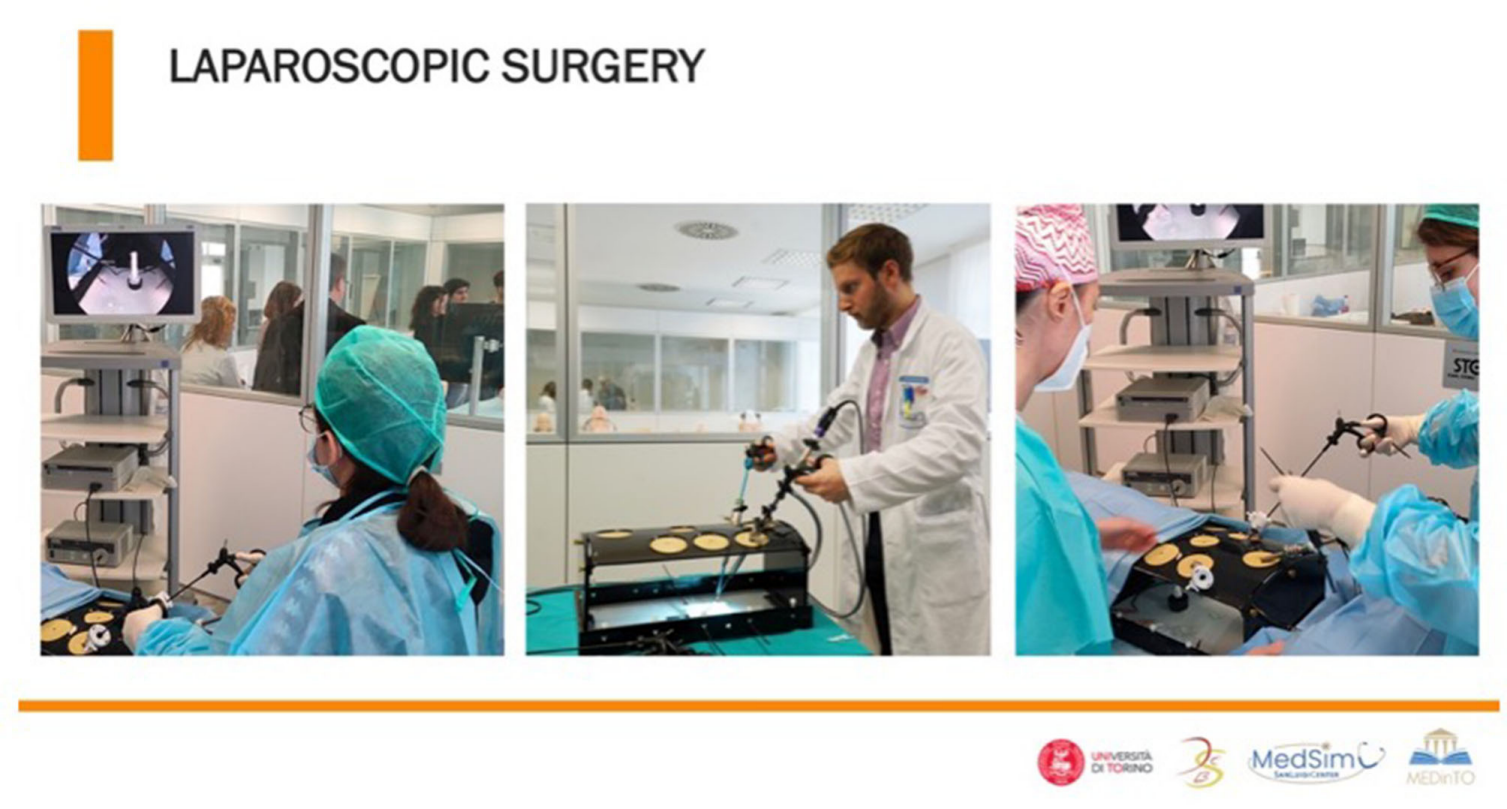
Laparoscopic surgery training in the simulation environment.

In the last few years, both surgery and surgical education have undergone a dramatic evolution with the advent of minimally invasive surgery. In this field, laparoscopy is a surgical procedure done through one or more small incisions, using small tubes, cameras, and surgical instruments. This approach is technically complex, as it requires both classical surgical skills and psychomotor skills associated with minimally invasive surgeries (e.g., good hand–eye coordination) (64). Training simulators, such as laparoscopic pelvic box trainers, have therefore been developed for different surgeries (urology, gynecology, etc.) (Figure 2). These learning resources could be extremely useful for students to face for the first time the required dexterity and psychomotor skills that characterize surgery. Without the use of a simulative environment, experiencing the fundamental surgical steps would only be possible by participating in a surgical residency program or performing surgical procedures on patients (65).

## DoNTStoptraining

**Figure 3:**
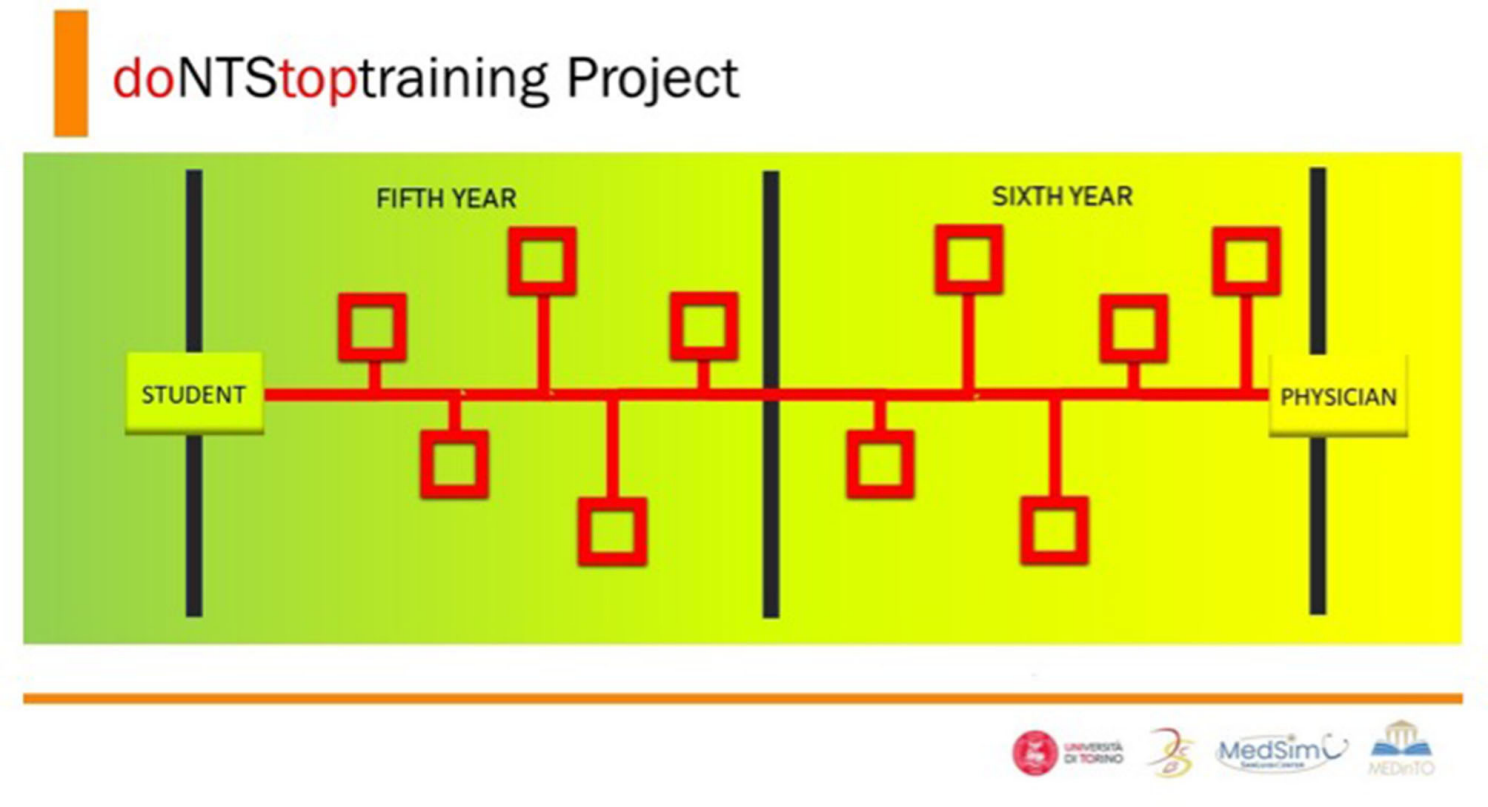
DoNTStoptraining project is a network of simulation-based events with the goal of training students in proficiency for managing social and cognitive skills essential to becoming a physician focused on patient safety and appropriate self-management from the first day of professional practice.

Medical schools aim to provide the best possible medical education, but do they train students to become physicians? The day after they graduate, medical students should switch from an educational to a professional mindset. Is it something they can accomplish in a matter of hours? MedInTo project “DoNTStoptraining” sets up a network of simulation-based events, with the goal of training students in proficiency for managing social and cognitive skills required to become physicians focused on patient safety and appropriate self-management from the first day of professional practice (Figure 3). While the early introduction of simulation in the medical curriculum brings numerous benefits, it is important to strike a balance between simulation and clinical exposure. Simulation should supplement, not replace, real-life patient care experiences. Integrating simulation strategically throughout the curriculum ensures that students receive a comprehensive education that prepares them for clinical practices.

## “Introduction to taking care” clerkship

We recently reported the results from a sentiment analysis of the logbooks written by the first-year students of the degree program. These students participated in the interprofessional education (IPE) clerkship “Introduction to Taking Care.” The IPE involved first-year MD students in tandem with third-year nursing students (Academic Year 2021-2022). The IPE included different teaching activities, such as the ability to practice a venipuncture, female urinary catheterization, the ability to check arterial blood pressure.

In the teaching location, MD students were supported by the third-year nursing students, who acted as peer coachers, under the supervision of a clinical tutor.

Our results support the finding that the utilization of an *Advanced Medical Simulation Centre (AMSC)* was frequently linked to positive sentiments. In this setting, the sentiment analysis of the logbooks showed that positive sentiments including “improvement,” “help,” and “satisfied” were frequently reported in association with the AMSC (66).

## Clinical exposure vs. simulation training: cost-effective considerations

By definition, the simulation program should not replace clinical exposure. The available data on its effectiveness derive from limited experience on several specific tasks that use non-homogeneous assessment tools, often developed internally and not previously validated (67–69). The effectiveness also depends on the students' willingness to feel fully involved in the proposed clinical scenario, without underestimating the almost realistic nature of the situation. Simulated training supports students in acquiring new skills, particularly for their initial interactions with patients at the beginning of their careers and for the acquisition of clinical practice in subsequent years. However, the relationship with patients and their clinical and behavioral complexity cannot be replaced with simulated training either during visits or during diagnostic or therapeutic decisions. The simulation program prepares them, increasing their awareness.

## Standardization and assessment: points to consider

The process of standardizing medical curriculum and examinations is a multifaceted and continuous endeavor with the objective of ensuring that medical education remains up-to-date, rigorous, and pertinent. The subject matter encompasses various essential elements, as detailed in Table 1. It is essential to acknowledge that the standardization of medical education is a dynamic undertaking that necessitates flexibility in response to evolving medical practices and healthcare demands. However, with technological advancements and new education approaches arising, including simulation training, international efforts involving different stakeholders are imperative to improve standardization, consistency, and comparability of the medical curricula.

**Table 1:** Standardization and assessment: points to consider.

**Implementation of a competency-based approach**	**The implementation of a competency-based approach in medical education is advocated, wherein the curriculum is structured around clearly delineated competencies and desired learning outcomes. Competency-based education places emphasis on the practical skills and abilities that students should possess, rather than solely focusing on their acquisition of knowledge. This feature enables adaptability in the development of educational curricula**.
The significance of accreditation standards	Accreditation bodies assume a pivotal role in the establishment of standardized medical education. To assure the quality and consistency of medical programs, standards are established that institutions must adhere to.
Curriculum design	It is imperative for medical schools to have a meticulously organized curriculum that encompasses fundamental subjects such as basic sciences, clinical skills, and professional growth. Curriculum committees frequently engage in the periodic evaluation and revision of curriculum to integrate emerging information and optimal methodologies.
Core competencies	The term, “core competencies,” refers to the fundamental abilities and skills that are expected of all medical graduates. These competencies encompass various areas, including effective communication, sound clinical reasoning, and adherence to ethical principles in practice. These competencies are fundamental to the process of curriculum design.
The incorporation of evidence-based procedures	The incorporation of evidence-based procedures should be prioritized in medical education. The integration of contemporary research findings and adherence to established clinical recommendations are of paramount importance to guarantee that students are acquiring the most up-to-date knowledge.
Clinical exposure and experience	It is imperative to guarantee that students are provided with sufficient opportunities for clinical exposure and experience. Clinical rotations, clerkships, and internships play a crucial role in the acquisition of practical skills and experiential knowledge.
Procedures of assessment	The standardization of assessment procedures is crucial to accurately measure the student performance. The assessment methods encompassed in this category consist of written examinations, evaluations of practical abilities, objective structured clinical examinations (OSCEs), and assessments pertaining to professionalism.
National licensing examinations	National licensing examinations are implemented in numerous countries as a means of guaranteeing that graduates possess a baseline level of proficiency. The examinations are frequently revised to align with advancements in medical knowledge and the implementation of new practices. This implementation should be taken into account when curricula are designed.
Enhancement of faculty development	Offer continuous chances for professional development to faculty members to ensure their currency in medical knowledge and pedagogical techniques.
Implementation of continuous improvement	It is imperative for medical schools to have a process that facilitates ongoing enhancement and refinement. Systematically gathering feedback from both current students and alumni can be a valuable practice in identifying potential areas for improvement within the program.
Promotion of interprofessional education	The promotion of interprofessional education is advocated to equip students with the necessary skills to effectively collaborate with other healthcare professionals, thereby mirroring the dynamics of real-world healthcare environments.
Attention to diversity and marginalized communities	The integration of training programs focused on cultural competence, health disparities, and diversity is crucial in equipping students with the necessary skills to effectively cater to various patient populations.
Integration of a global health perspective	Integrate a global health perspective into the curriculum to equip students with the necessary knowledge and skills to address healthcare and public health concerns in a global context.
Telemedicine and digital health technology	The integration of telemedicine and digital health technology is imperative in the contemporary digital era, necessitating the inclusion of corresponding training.
Ethical considerations	It is imperative to ensure that the principles of ethics and professionalism are taught and tested with a high level of rigor.

For accomplishing this objective, it is essential to establish a continuous partnership among medical educational institutions, accrediting entities, healthcare organizations, and regulatory agencies.

## Assessment of long-term retention following the simulation-based training

The main objective of education is to facilitate the enduring acquisition of knowledge and/or skills (70). In the realm of medical education, physicians undergo training to effectively transfer and adapt their knowledge to a diverse range of forthcoming clinical difficulties, drawing upon a well-preserved expertise. As stated earlier, numerous studies have provided evidence about the efficacy of incorporating simulation into the medical curriculum as a means to enhance knowledge, skills, and behaviors (71–74).

Nevertheless, evaluating the extent to which memory is retained following the training for intricate technical skills poses a challenging task (70). A meta-analysis conducted in 2013 examined the use of simulation in pediatric training and identified a lack of educational simulation models that are essential for the acquisition and retention of skills. This scarcity can be attributed to the limited number of comparative studies conducted in medicine when specifically targeting the retention of acquired skills and knowledge (75). Despite the increase in data on simulation-based training in medicine, many of these new studies use a “no intervention group” as a control and poorly define the timing and frequency for repeating the simulation sessions (75). Moreover, there is heterogeneity in re-training recommendations (76–78). It is part of the MedInTO mission to assess long-term retention of the skills acquired after the simulation training and to determine factors that can influence this pedagogical process (Table 2) (76).

**Table 2:** Pillars of long-term retention of the skills acquired after simulation training.

**Follow-up assessments**	**Schedule follow-up assessments at regular intervals (e.g., 6 months, 1 year, and 2 years) after the initial simulation training to gauge the retention of knowledge and skills**.
Comparison with control groups	Include control groups of individuals who did not receive simulation training to compare their performance with those who did. This allows for a more robust assessment of long-term retention.
Standardized assessments	Use standardized and validated assessment tools to measure the retention of specific knowledge and skills taught during simulation training.
Clinical performance	Evaluate participants' clinical performance in real patient care settings to assess the application of skills learned through simulation training.
Self-assessment and reflection	Encourage participants to self-assess their knowledge and skills periodically and reflect on their performance in real clinical scenarios. Self-assessment can provide insights into perceived retention.
Feedback and debriefing	Conduct feedback sessions and debriefing with participants to discuss their experiences and any challenges faced when applying simulation-trained skills in practice.
Longitudinal studies	Consider conducting longitudinal studies that track participants' performance and patient outcomes over an extended period to assess the sustained impact of simulation training.
Maintenance training	Offer maintenance or refresher training sessions to reinforce and update the knowledge and skills acquired during the initial simulation training.
Peer review and feedback	Encourage peer review and feedback among healthcare professionals to provide insights into the retention and application of simulation-based training in clinical practice.
Patient outcomes	Assess the impact of simulation-based training on patient outcomes, such as reduced complications, improved recovery rates, or enhanced patient satisfaction, over the long term.

## Conclusion

Simulation in medicine is increasingly available, portable, and advanced.

Is an early introduction into medical curricula feasible? Our experience at the MD program in Medicine and Surgery of University of Turin-MedInTo supports our team in pursuing this goal. Continued efforts are required to improve, with a particular focus on non-technical skills, and standardize curricula and assessments.

## Data availability statement

The original contributions presented in the study are included in the article/Supplementary material, further inquiries can be directed to the corresponding author.

## Ethics statement

Ethical review and approval was not required for the study on human participants in accordance with the local legislation and institutional requirements. Written informed consent was not required to participate in this study in accordance with the local legislation and institutional requirements. No animal was involved in this study.

## Author contributions

DL: Conceptualization, Data curation, Methodology, Writing—original draft, Writing—review & editing. FA: Conceptualization, Validation, Visualization, Writing—original draft, Writing—review & editing. CC: Conceptualization, Visualization, Writing—original draft, Writing—review & editing. DG: Conceptualization, Visualization, Writing—original draft, Writing—review & editing. MM: Conceptualization, Visualization, Writing—original draft. LOd: Methodology, Visualization, Writing—original draft, Writing—review & editing. LOs: Conceptualization, Visualization, Writing—original draft, Writing—review & editing. PP: Conceptualization, Writing—review & editing, Data curation, Formal analysis, Supervision. SR: Conceptualization, Writing—original draft, Writing—review & editing, Visualization. GR: Conceptualization, Data curation, Formal analysis, Supervision, Writing—review & editing. SS: Conceptualization, Data curation, Methodology, Writing—original draft, Writing—review & editing.

## Supplementary material

Supplementary Video The “Simulation circuit” as part of the simulation for emergency medical care for students' activity. In both the center and nearby environments, four contemporaneously simulative stations were organized.

## References

[1] McGaghie WC Barsuk JH Wayne DB Issenberg SB. Powerful medical education improves health care quality and return on investment. Med Teach. (2023) 6:1–13. 10.1080/0142159X.2023.2276038 37930940

[2] Lee J Kim H Kron F. Virtual education strategies in the context of sustainable health care and medical education: a topic modelling analysis of four decades of research. Med Educ. (2023). 10.1111/medu.15202 [Epub ahead of print]. 37794709

[3] Cardoso SA Suyambu J Iqbal J Jaimes DCC Amin A Sikto JT . Exploring the role of simulation training in improving surgical skills among residents: a narrative review. Cureus. (2023) 15:e44654. 10.7759/cureus.44654 37799263 PMC10549779

[4] Pietersen PI Hertz P Olsen RG Møller LB Konge L Bjerrum F . Transfer of skills between laparoscopic and robot-assisted surgery: a systematic review. Surg Endosc. (2023). 10.1007/s00464-023-10472-5 [Epub ahead of print]. 37875694

[5] Ritchie A Pacilli M Nataraja RM. Simulation-based education in urology - an update. Ther Adv Urol. (2023) 15:17562872231189924. 10.1177/17562872231189924 37577030 PMC10413896

[6] Ziv Stephen DS. Patient safety and simulation-based medical education. Med Teach. (2000) 22:489–95. 10.1080/01421590050110777 21271963

[7] Roberts S Desai A Checcucci E Puliatti S Taratkin M Kowalewski KF . Augmented reality” applications in urology: a systematic review. Minerva Urol Nephrol. (2022) 74:528–37. 10.23736/S2724-6051.22.04726-7 35383432

[8] Wanderling C Saxton A Phan D Sheppard L Schuler N Ghazi A. Recent advances in surgical simulation for resident education. Curr Urol Rep. (2023) 24:491–502. 10.1007/s11934-023-01178-1 37736826

[9] Porterfield JR Jr Podolsky D Ballecer C Coker AM Kudsi OY Duffy AJ . Structured resident training in robotic surgery: recommendations of the robotic surgery education Working Group. J Surg Educ. (2023). 10.1016/j.jsurg.2023.09.006 [Epub ahead of print]. 37827925

[10] Howard T Iyengar KP Vaishya R Ahluwalia R . High-fidelity virtual reality simulation training in enhancing competency assessment in orthopaedic training. Br J Hosp Med. (2023) 84:1–8. 10.12968/hmed.2022.0360 37769263

[11] Glossop SC Bhachoo H Murray TM Cherif RA Helo JY Morgan E . Undergraduate teaching of surgical skills in the UK: systematic review. BJS Open. (2023) 7:zrad083. 10.1093/bjsopen/zrad083 37819804 PMC10566575

[12] Hamad NB Folorunsho EF. Simulated participants' experiences and challenges with online and face-to-face interactions during COVID-19: a case study in UAEU. Simul Healthc. (2023). 10.1097/SIH.0000000000000752 37823744

[13] Kube P Levy C Diaz MCG Dickerman M. Improving the procedure of delivering serious news: impact of a six-month curriculum for second year pediatric residents. Am J Hosp Palliat Care. (2023). 10.1177/10499091231206562 [Epub ahead of print]. 37822065

[14] Sizemore J Bailey A Sankineni S Clark K Manivannan S Kolar M . Training to transition: using simulation-based training to improve resident physician confidence in hospital discharges. MededPortal. (2023) 19:11348. 10.15766/mep_2374-8265.11348 37720418 PMC10502193

[15] Song SY Choi WK Kwak S. A model study for the classification of high-risk groups for cardiac arrest in general ward patients using simulation techniques. Medicine. (2023) 102:e35057. 10.1097/MD.0000000000035057 37713881 PMC10508528

[16] Tayeb BO Shubbak FA Doais KS Yamani AN Dhaifallah DG Alsayed EF . Uses of simulation-based education for anesthesiology training, certification and recertification: a scoping review. J Taibah Univ Med Sci. (2023) 18:1118–23. 10.1016/j.jtumed.2023.03.015 37881638 PMC10594003

[17] Matlala S. Educators' perceptions and views of problem-based learning through simulation. Curationis. (2021) 44:e1–7. 10.4102/curationis.v44i1.2094 33764129 PMC8008084

[18] Chanda A Ruchti T Unnikrishnan V. Computational modeling of wound suture: a review. IEEE Rev Biomed Eng. (2018) 11:165–76. 10.1109/RBME.2018.2804219 29994368

[19] Morris MC Gallagher TK Ridgway PF. Tools used to assess medical students competence in procedural skills at the end of a primary medical degree: a systematic review. Med Educ Online. (2012) 17. 10.3402/meo.v17i0.18398 PMC3427596 22927716

[20] Kodikara K Seneviratne T Premaratna R. Pre-clerkship procedural training in venipuncture: a prospective cohort study on skills acquisition and durability. BMC Med Educ. (2023) 23:729. 10.1186/s12909-023-04722-2 37803328 PMC10559527

[21] Vitale KM Barsuk JH Cohen ER Wayne DB Hansen RN Williams LM . Simulation-based mastery learning improves critical care skills of advanced practice providers. ATS Sch. (2023) 4:48–60. 10.34197/ats-scholar.2022-0065OC 37089675 PMC10117416

[22] Liu S Watkins K Hall CE Liu Y Lee S-H Papandria D . Utilizing simulation to evaluate robotic skill acquisition and learning decay. Surg Laparosc Endosc Percutan Tech. (2023) 33:317–23. 10.1097/SLE.0000000000001177 37235716

[23] Santana BS Paiva AAM Magro M. Skill acquisition of safe medication administration through realistic simulation: an integrative review. Rev Bras Enferm. (2020) 73:e20190880. 10.1590/0034-7167-2019-0880 33338159

[24] Almoghirah H Illing J Nazar M Nazar H. A pilot study evaluating the feasibility of assessing undergraduate pharmacy and medical students interprofessional collaboration during an online interprofessional education intervention about hospital discharge. BMC Med Educ. (2023) 23:589. 10.1186/s12909-023-04557-x 37605168 PMC10441699

[25] Armijo-Rivera S Ferrada-Rivera S Aliaga-Toledo M Pérez LA. Application of the Team Emergency Assessment Measure Scale in undergraduate medical students and interprofessional clinical teams: validity evidence of a Spanish version applied in Chile. Front Med. (2023) 10:1256982. 10.3389/fmed.2023.1256982 37771978 PMC10525305

[26] Radcliffe E Servin R Cox N Lim S Tan QY Howard C . What makes a multidisciplinary medication review and deprescribing intervention for older people work well in primary care? A realist review and synthesis. BMC Geriatr. (2023) 23:591. 10.1186/s12877-023-04256-8 37743469 PMC10519081

[27] Donato Z Syros A Milner J Pandya S Tandron M Hernandez G. “Sawbones”: A pilot study assessing simulation-based orthopedic training for medical students. J Orthop. (2023) 44:66–71. 10.1016/j.jor.2023.08.012 37700780 PMC10493496

[28] Kolbe M Goldhahn J Useini M Grande B. “Asking for help is a strength”-how to promote undergraduate medical students' teamwork through simulation training and interprofessional faculty. Front Psychol. (2023) 14:1214091. 10.3389/fpsyg.2023.1214091 37701867 PMC10494543

[29] Liaw SY Ooi SW Rusli KDB Lau TC Tam WWS Chua WL . Nurse-physician communication team training in virtual reality versus live simulations: randomized controlled trial on team communication and teamwork attitudes. J Med Internet Res. (2020) 22:e17279. 10.2196/17279 32267235 PMC7177432

[30] Lorenzini G Zamboni A Gelati L Di Martino A Pellacani A Barbieri N . Emergency team competencies: scoping review for the development of a tool to support the briefing and debriefing activities of emergency healthcare providers. J Anesth Analg Crit Care. (2023) 3:24. 10.1186/s44158-023-00109-3 37507807 PMC10386683

[31] Charles F . Evaluation of practices through simulation: Implementation of Horror Week in a cytotoxic preparation unit. Ann Pharm Fr. (2023) 81:1099–108. 10.1016/j.pharma.2023.07.007 37541617

[32] Lamberta M Aghera A. Latent Safety Threat Identification via Medical Simulation. Treasure Island, FL: StatPearls (2023). 31751099

[33] Krautscheid LC. Embedding microethical dilemmas in high-fidelity simulation scenarios: preparing nursing students for ethical practice. J Nurs Educ. (2017) 56:55–8. 10.3928/01484834-20161219-11 28118477

[34] Dias R Robinson K Poirier P. The effect of simulation on nursing student perceptions of readiness to provide end-of-life care. J Hosp Palliat Nurs. (2023) 25:E116–23. 10.1097/NJH.0000000000000979 37930167

[35] Lewis DY Stephens KP Ciak AD. QSEN curriculum integration and bridging the gap to practice. Nurs Educ Perspect. (2016) 37:97–100. 10.5480/14-1323 27209868

[36] Dahmen L Linke M Schneider A Kühl SJ. Medical students in their first consultation: a comparison between a simulated face-to-face and telehealth consultation to train medical consultation skills. GMS J Med Educ. (2023) 40:Doc63. 10.3205/zma001645 37881523 PMC10594035

[37] Takhdat K Rebahi H Rooney DM Babram MA Benali A Touzani S . The impact of brief mindfulness meditation on anxiety, cognitive load, and teamwork in emergency simulation training: a randomized controlled trial. Nurse Educ Today. (2023) 132:106005. 10.1016/j.nedt.2023.106005 37944276

[38] Gotz K. Explaining the health system in a practical way - the use of a simulation game in medical sociology teaching. GMS J Med Educ. (2023) 40:Doc57. 10.3205/zma001639 37881520 PMC10594036

[39] Liew SC Tan MP Breen E Krishnan K Sivarajah I Raviendran N . Microlearning and online simulation-based virtual consultation training module for the undergraduate medical curriculum - a preliminary evaluation. BMC Med Educ. (2023) 23:796. 10.1186/s12909-023-04777-1 37880711 PMC10601318

[40] Sun L Liu D Lian J Yang M. Application of flipped classroom combined with virtual simulation platform in clinical biochemistry practical course. BMC Med Educ. (2023) 23:771. 10.1186/s12909-023-04735-x 37845661 PMC10577961

[41] Babar ZUD Max SA Martina BG Rosalia RA Peek JJ van Dijk A . Virtual reality simulation as a training tool for perfusionists in extracorporeal circulation: establishing face and content validity. JTCVS Tech. (2023) 21:135–48. 10.1016/j.xjtc.2023.06.004 37854847 PMC10579814

[42] Efe IE Çinkaya E Kuhrt LD Bruesseler MMT Mührer-Osmanagic A. Neurosurgical education using cadaver-free brain models and augmented reality: first experiences from a hands-on simulation course for medical students. Medicina. (2023) 59. 10.3390/medicina59101791 37893509 PMC10608257

[43] Morrison TM Stitzel JD Levine SM. Modeling and simulation in biomedical engineering: regulatory science and innovation for advancing public health. Ann Biomed Eng. (2023) 51:1–5. 10.1007/s10439-022-03116-7 36562847

[44] Kasaie P Kelton WD Ancona RM Ward MJ Froehle CM Lyons MS. Lessons learned from the development and parameterization of a computer simulation model to evaluate task modification for health care providers. Acad Emerg Med. (2018) 25:238–49. 10.1111/acem.13314 28925587 PMC5880547

[45] Dau L Almeida PA Kulcheski AL Milcent PA Filho ES. Construct validity and experience of using a low-cost arthroscopic shoulder surgery simulator. Rev Bras Ortop. (2023) 58:e790–7. 10.1055/s-0043-1771003 37908521 PMC10615612

[46] Lord S Geary S Lord G. Application of a low-cost, high-fidelity proximal phalangeal dislocation reduction model for clinician training. West J Emerg Med. (2023) 24:839–46. 10.5811/WESTJEM.59471 37788023 PMC10527832

[47] Ladowski JM . A novel low-cost model of superficial abscess for trainee education in incision and drainage. Surg Open Sci. (2023) 14:124–7. 10.1016/j.sopen.2023.07.015 37593672 PMC10428102

[48] Lerner JS Li Y Valdesolo P Kassam KS. Emotion and decision making. Annu Rev Psychol. (2015) 66:799–823. 10.1146/annurev-psych-010213-115043 25251484

[49] Habicht JL Kiessling C Winkelmann A. Bodies for anatomy education in medical schools: an overview of the sources of cadavers worldwide. Acad Med. (2018) 93:1293–300. 10.1097/ACM.0000000000002227 29561275 PMC6112846

[50] Said Ahmed MAA. Use of the anatomage virtual table in medical education and as a diagnostic tool: an integrative review. Cureus. (2023) 15:e35981. 10.7759/cureus.35981 37041931 PMC10083048

[51] Calì C Nuzzolese E. The use of the Anatomage Table for improving forensic odontology education and training. Ann 3D Print Med. (2022) 7:100073. 10.1016/j.stlm.2022.100073

[52] Co M Chiu S Billy Cheung HH. Extended reality in surgical education: a systematic review. Surgery. (2023) 174:1175–83. 10.1016/j.surg.2023.07.015 37640664

[53] Carlson CG. Virtual and augmented simulations in mental health. Curr Psychiatry Rep. (2023) 25:365–71. 10.1007/s11920-023-01438-4 37624512

[54] Holopainen R Tiihonen J Lahteenvuo M. Efficacy of immersive extended reality (XR) interventions on different symptom domains of schizophrenia spectrum disorders. A systematic review. Front Psychiatry. (2023) 14:1208287. 10.3389/fpsyt.2023.1208287 37599868 PMC10436301

[55] Randazzo G Reitano G Carletti F Iafrate M Betto G Novara G . Urology: a trip into metaverse. World J Urol. (2023) 41:2647–57. 10.1007/s00345-023-04560-3 37552265 PMC10582132

[56] Taghian A Abo-Zahhad M Sayed MS Abd El-Malek AH. Virtual and augmented reality in biomedical engineering. Biomed Eng Online. (2023) 22:76. 10.1186/s12938-023-01138-3 37525193 PMC10391968

[57] Amparore D Pecoraro A Checcucci E Cillis SD Piramide F Volpi G . 3D imaging technologies in minimally invasive kidney and prostate cancer surgery: which is the urologists' perception? Minerva Urol Nephrol. (2022) 74:178–85. 10.23736/S2724-6051.21.04131-X 33769019

[58] Amparore D Piramide F De Cillis S Verri P Piana A Pecoraro A . Robotic partial nephrectomy in 3D virtual reconstructions era: is the paradigm changed? World J Urol. (2022) 40:659–70. 10.1007/s00345-022-03964-x 35191992

[59] Dyer E Swartzlander BJ Gugliucci MR. Using virtual reality in medical education to teach empathy. J Med Libr Assoc. (2018) 106:498–500. 10.5195/jmla.2018.518 30271295 PMC6148621

[60] Pottle J. Virtual reality and the transformation of medical education. Fut Healthc J. (2019) 6:181–5. 10.7861/fhj.2019-0036 31660522 PMC6798020

[61] Liu Z Chen Q Wu J Li X He Y Yu Q. Simulation-based training in asthma exacerbation for medical students: effect of prior exposure to simulation training on performance. BMC Med Educ. (2022) 22:223. 10.1186/s12909-022-03300-2 35361196 PMC8973632

[62] Nuzzo A Tran-Dinh A Courbebaisse M Peyre H Plaisance P Matet A . Improved clinical communication OSCE scores after simulation-based training: Results of a comparative study. PLoS ONE. (2020) 15:e0238542. 10.1371/journal.pone.0238542 32886733 PMC7473530

[63] Scheepers RA Emke H Epstein RM Lombarts KMJMH. The impact of mindfulness-based interventions on doctors' well-being and performance: a systematic review. Med Educ. (2020) 54:138–49. 10.1111/medu.14020 31868262 PMC7003865

[64] Clayman RV Kavoussi LR Soper NJ Dierks SM Meretyk S Darcy MD . Laparoscopic nephrectomy: initial case report. J Urol. (1991) 146:278–82. 10.1016/S0022-5347(17)37770-4 1830346

[65] Somani BK Van Cleynenbreugel B Gözen A-S Skolarikos A Wagner C Beatty J . Outcomes of European Basic Laparoscopic Urological Skills (EBLUS) Examinations: results from European School of Urology (ESU) and EAU Section of Uro-Technology (ESUT) over 6 Years (2013-2018). Eur Urol Focus. (2020) 6:1190–4. 10.1016/j.euf.2019.01.007 30661943

[66] Versino E. Introduction to taking care' clerkship at the degree in medicine & surgery programme in Orbassano (University of Torino). Med Nei Secoli. (2023) 1.

[67] Clemens L. The efficacy and cost-effectiveness of a simulation-based primary care procedural skills training program for advanced practice providers. J Contin Educ Health Prof . (2023). 10.1097/CEH.0000000000000530 [Epub ahead of print]. 37713161

[68] Ng DS Yip BHK Young AL Yip WWK Lam NM Li KK . Cost-effectiveness of virtual reality and wet laboratory cataract surgery simulation. Medicine. (2023) 102:e35067. 10.1097/MD.0000000000035067 37800761 PMC10552957

[69] Signorini S Imberti L Pirovano S Villa A Facchetti F Ungari M . Intrathymic restriction and peripheral expansion of the T-cell repertoire in Omenn syndrome. Blood. (1999) 94:3468–78. 10.1182/blood.V94.10.3468.422k34_3468_3478 10552957

[70] Boet S Granry JC Savoldelli G. La simulation en santé: de la théorie à la pratique. (France: Springer Verlag) (2013). 1 p.

[71] Miyasaka KW Martin ND Pascual JL Buchholz J Aggarwal R. A simulation curriculum for management of trauma and surgical critical care patients. J Surg Educ. (2015) 72:803–10. 10.1016/j.jsurg.2015.03.001 25921186 PMC4540678

[72] Steadman RH Huang YM. Simulation for quality assurance in training, credentialing and maintenance of certification. Best Pract Res Clin Anaesthesiol. (2012) 26:3–15. 10.1016/j.bpa.2012.01.002 22559952

[73] Wayne DB DA Feinglass J Fudala MJ Barsuk JH McGaghie WC. Simulation-based education improves quality of care during cardiac arrest team responses at an academic teaching hospital: a case-control study. Chest. (2008) 133:6. 10.1378/chest.07-0131 17573509

[74] Letcher DC Varenhorst LJ. Simulation-based learning: improving knowledge and clinical judgment within the NICU. Clin Simul Nurs. (2017) 13:6. 10.1016/j.ecns.2017.03.001

[75] Cheng A Lang TR Starr SR Pusic M Cook DA. Technology-enhanced simulation and pediatric education: a meta-analysis. Pediatrics. (2014) 133:e1313–23. 10.1542/peds.2013-2139 24733867

[76] American Heart Association (AHA). AHA Program Administration Manual. 6th ed. US Version (2018). Available online at: http://www.lifesupporttraining.org/sites/default/files/aha_pam.pdf (accessed November 17, 2023).

[77] Resuscitation Council (UK) (2017). Available online at: https://www.resus.org.uk (accessed November 17, 2023).

[78] French Society of Pediatrics. ERC Courses (2018). Available online at: http://www.sfpediatrie.com/page/formations-erc (accessed November 17, 2023).

